# *Tinospora cordifolia* chloroform extract inhibits LPS-induced inflammation via NF-κB inactivation in THP-1cells and improves survival in sepsis

**DOI:** 10.1186/s12906-021-03244-y

**Published:** 2021-03-20

**Authors:** Sheena Philip, Greeshma Tom, Padmaja Balakrishnan Nair, Sankar Sundaram, Asha Velikkakathu Vasumathy

**Affiliations:** 1 Plant Based Bioactives and Disease Biology Laboratory, Rajiv Gandhi Centre for Biotechnology, Thiruvananthapuram, 695014 India; 2 University of Kerala, Thiruvananthapuram, India; 3 Ayurveda Research Institute for Mother and Child Health Care, Thiruvananthapuram, India; 4 Department of Pathology, Government Medical College, Kottayam, India

**Keywords:** *Tinospora cordifolia*, THP-1, NF-κB, Endotoxemia, TNF-α

## Abstract

**Background:**

*Tinospora cordifolia* (Willd).Miers is a perennial climbing medicinal shrub that has been traditionally used for the treatment of chronic inflammatory ailments. Our previous pre- clinical studies on anti-inflammatory effects, proved that the chloroform extract of *T. cordifolia* (CETC) suppressed the LPS induced up-regulation of pro-inflammatory biomarkers, hence, further follow up study was carried out to evaluate whether CETC can exhibit a protective effect against LPS induced lethal endotoxemia in vivo and also to analyze the impact of CETC pre-treatment on the secretion of pro-inflammatory cytokines in vitro by THP-1 cells.

**Methods:**

To corroborate our previous preclinical studies on inflammation, we investigated the mechanism of the anti-inflammatory effect of *T. cordifolia* on THP-cells which were pre-incubated with CETC (30 min) and stimulated subsequently with LPS (1 μg/ml) for 20 h. Levels as well as gene expressions of various cytokines were compared with that of LPS alone incubated cells. Alongside, in vivo oral anti-inflammatory efficacy against LPS induced endotoxemia study was effectuated, wherein rats were administered with CETC 48, 24, 12 and 1 h prior to the injection of LPS and the survival of rats were monitored upto 10 days. Cytokine levels were quantified by ELISA. Nitrite levels were measured using Griess reagent. Expression of pro-inflammatory proteins was inspected in rat tissues by histochemical and immuno -histochemical examinations.

**Results:**

CETC was able to down-regulate the up-regulation of pro-inflammatory biomarkers in THP-1 macrophages though blockade of NF-κB nuclear translocation and could improve the survival rate during endotoxemic episodes with a marked suppression of the tissue expression of pro-inflammatory proteins.

**Conclusion:**

These findings concomitantly reveal the anti-inflammatory mechanism of CETC and support us to move forward for the development of drugs against disorders resulting from deregulated immune reactions.

**Supplementary Information:**

The online version contains supplementary material available at 10.1186/s12906-021-03244-y.

## Background

The topic of inflammation continues to seize the global scientific research community’s attention, undeniably owing to its implication in virtually all diseases which affect living beings [1]. Inflammation is defined as a complex, fundamental response utilized by the immune system for the purpose of countering the effects of noxious stimuli, including pathogenic microbes, irritant chemicals or toxins, damaged cells or tissues and tumor growth. Although it is acknowledged that inflammatory responses’ nature and type might vary considerably among different disease states, they are generally characterised by the improper regulation of a common set of genes, particularly those which fit under the heading of cytokines-encoding varieties. Because an inflammatory response that transmits the inflammatory stimuli to other cell types and tissues involves a variety of mediators and multiple-signalling pathways, it is impossible to focus on any one specific area in the process of treating it. Therefore, an effective proven strategy to control inflammatory reactions is controlling the production or function of cytokines [2, 3].

Multiple studies have corroborated the hypothesis that NF-κB activation occurring in the cytoplasm in response to inflammatory stimuli arising at the cell surface makes up a crucial pathway that helps to upregulate the pro-inflammatory genes of the nucleus. For this reason, NF-κB and the signaling cascades that regulate its function have become a prime focus in efforts oriented towards the discovery of anti-inflammatory drugs. By reason of its being attributed to the incidence of a large number of chronic inflammatory ailments in which improper control over NF-κB activation has been noticed or is speculated to exist, such efforts appear to be well justified [4, 5].

Sepsis is to be identified as an exaggerated systemic inflammatory response, a phenomenon owing its origins to systemic infection and continuing to hold the unfortunate status of a major cause of mortality around the globe. The most common pathogenic factor under consideration in this matter is one’s exposure to LPS, a structural component of Gram-negative bacteria. Sepsis is characterised by hypotension, vasoplegia and hypoperfusion of the major organs, all possessing potential of causing multiple organ failure and by extension, ultimately, death. Although many treatment modalities against sepsis have already been introduced, the sought-out breakthrough, promising a reliable solution to bring about a definitive, substantial improvement with regard to the mortality rate remains lacking thus far [6].

It is to be noted that detailed investigations regarding the pathophysiology of sepsis have evidenced that microorganisms themselves do not, in fact, induce multiple organ failure. It is rather the case that infection triggers an inflammatory response in the host which causes vascular system damage, intravascular coagulation, and tissue destruction which, in turn, cause progressive organ dysfunction. The sustained systemic inflammatory response, accompanied by the massive secretion of pro-inflammatory cytokines, has been regarded as the central pathophysiological mechanism behind the development of multiple organ failure in septic shock [7]. This emphasis upon unregulated and amplified inflammatory response as a factor behind multiple organ failure has inspired numerous clinical trials which aimed to block several pro-inflammatory signalling cascades in sepsis’ early phase. Most of the clinical signs observed in sepsis can be replicated in in vivo models through the injection of bacterial components such as LPS. Thus, lethal endotoxemia has been widely approved as an experimental model of Gram-negative endotoxic shock [8].

Medicinal plants are known to contain a multitude of bioactive molecules which could potentially be harnessed for the development of novel therapeutically-active formulations or compounds which would have superior effectiveness while carrying fewer side effects. Furthermore, herbal remedies continue to constitute an essential resource for the discovery of modern drugs [9]. For example, one could consider *Tinospora cordifolia* (Willd.) Miers, which belongs to the family Menispermaceae and is indigenous to the tropical areas within the nation of India. In the present study, the aim was to investigate the efficacy of Chloroform Extract of *T. cordifolia* (CETC) in protecting rats from lethal endotoxemia. Furthermore, we sought to determine whether the potent CETC could abolish the LPS-aided upregulation of pro-inflammatory cytokines in the human macrophage cell line, THP-1. For the abovementioned purposes we have used LPS-primed rat model as well as LPS activated THP-1 cell lines.

## Methods

### Chemicals

Fetal bovine serum (FBS), Lipopolysaccharide, sodium dodecyl sulphate (SDS), 3-(4, 5-dimethylthiazol- 2-yl)-2, 5-diphenyl tetrazolium bromide (MTT), and Bradford reagent were purchased from Sigma, USA. The antibiotic–antimycotic mixture was acquired from Gibco-BRL, USA. Indomethacin was obtained from Cayman chemicals. All other chemicals used in this study were of analytical grade.

### Preparation plant extract

*T. cordifolia* was collected from Government Ayurveda College, Thiruvananthapuram. 10 g of dried plant powder was subjected to soxhlet extraction using chloroform, followed by the evaporation of the solvent under vacuum originating from a rotary evaporator. The percentage yield was determined to be 3.85%. A voucher specimen was kept in the institute herbarium (Ethno 42). Stock solutions (100 mg/ml) of *T. cordifolia* chloroform extract (CETC) were made in DMSO. Required dilutions were made at the time of the experiment in 10% RPMI medium.

### Cell culture

Human monocytic THP-1 cells procured from the American Type Culture Collection, Rockville, USA (THP-1Cells(ATCC^(R)^_TIB-202_^Tm^) were grown in RPMI-1640 medium containing 10% heat inactivated FBS (Gibco, GrandIsland, USA) and antibiotic–antimycotic mix solution by maintaining a steady temperature of 37^0^ C in a humidified, 5% CO2 atmosphere (Hera cell 150, Heraeus, Langenese, Germany).

### Nitrite determination

The Griess reaction was employed in order to determine the concentration of nitrite present in the culture media [10]. Equal volumes of Griess reagent (1% sulfanilamide, 0.1% naphtyl ethylene diamide in 2.5% phosphoric acid) and cell-free culture supernatants were mixed together and pipetted into the wells of an ELISA plate. They were then maintained in these conditions for a total of 10 min. The absorbance of the final product was determined spectrophotometrically at 525 nm using an ELISA plate reader. Additionally, the nitrite levels were determined from a sodium nitrite standard curve.

### Western blot analysis

THP-1 cells were pre-treated with CETC (50, 100 and 200 μg/ml) for 30 min and subsequently elicited with LPS (1 μg/ml) for 20 h at 37^0^ C. After the desired period of treatment, the total protein was isolated. Equal amounts of protein (quantified by Bradford’s method) from each sample were then resolved on a 12% SDS-Poly acrylamide gel (Mini-PROTEAN 3 Cell, Bio-Rad, Hercules, USA) and electro-blotted onto a PVDF membrane. After blocking with 5% BSA, the membrane was sequentially incubated with a primary antibody (Santa Cruz Biotechnology, USA) and an alkaline phosphatase-conjugated secondary antibody, followed by detection using BCIP/NBT premixed solution (Sigma-Aldrich, USA).

### Confocal laser scanning microscopy

THP-1 macrophages were directly grown on sterile glass cover slips kept in 6 well plates for 24 h. Following 1 hour of stimulation with 1 μg/ml LPS and/or indicated concentration of CETC, the cells were fixed in 3% cold paraformaldehyde in PBS, permeabilized with 0.25% Triton X-100, and blocked with 1% BSA. Fixed cells were then incubated (1 h) with monoclonal anti-rabbit IgG to NF-κB p65 (Five Photon Biochemicals, USA) followed by PBS washing. The cells were then incubated further with Alexa-fluor 488 conjugated goat anti-rabbit IgG (Abcam, USA). After two PBS washes, cover slips were mounted with glycerol-PBS (1:1) and photographed using a confocal microscope.

### Determination of PGE_2_, IL-1β and IL-6 by ELISA in cell culture supernatant

THP-1 cells were pre-treated for 30 min with CETC (50, 100 and 200 μg/ml) and stimulated subsequently with LPS (1 μg/ml) for 20 h. After incubation, the culture supernatant was collected and added to 96 well plates immobilized with PGE_2_, IL-1β or IL-6 antibodies (Abcam, Cambridge, USA). Absorbances were measured using a microplate reader.

### Cell viability assay

The cytotoxic potential of CETC was assessed by the conventional MTT assay, as described previously [11].

### In vivo endotoxin shock model

#### Grouping and treatment strategy

It is important to emphasize that all experiments involving the participation of animals were carried out in strict accordance with the guidelines of the Institutional Animal Ethics Committee (IAEC No.301/VVA/2015). Seventy-two male Charles Wistar rats (180-200 g) obtained from the institutional animal research facility were randomly assigned into six groups. The rats were then orally administered with CETC four times (i.e. 48, 24, 12 and 1 h) before the intraperitoneal injection of endotoxin. All drug or vehicle administrations were carried out using an oral cannula as follows:

**Table:** 

Groups	Treatment	Concentration	Number of rats
I	Saline (0.9%)	10 ml/ Kg body weight	6
2	Saline (0.9%) + LPS	10 ml/ Kg body weight	6
3	Indomethacin+ LPS	10 mg/Kg body weight	6
4	CETC + LPS	500 mg/Kg body weight	6
5	CETC + LPS	250 mg/Kg body weight	6
6	CETC + LPS	125 mg/Kg body weight	6

All rats were housed in poly-acrylic cages kept in the Institute’s Animal Research Facility and were fed with standard commercial laboratory chew (Sai Durga feeds and foods, Bangalore, India) and were provided with filtered water ad libitum. The cages were retained in adherence to the protocols dictating standard conditions (12 h’ light/dark cycle, 25 ± 3 °C and 60 ± 5% humidity) throughout the experimental period.

#### Rat survival rate analysis

After the oral administrations were carried out, all rats were returned to their cages and were provided with free access to water. 1 h post the final oral administration of the drug (CETC/indomethacin) or vehicle, each rat (except group 1 rats) was injected intra-peritoneally (i.p.) with LPS (15 mg/Kg body weight). Drug or vehicle administrations were carried out using an oral cannula. Indomethacin (Cayman Chemicals, USA) was used as the reference standard drug (10 mg/kg b.w). In an experimental group of 36 members, the survival of each rat was monitored for up to 10 days.

#### Histological analysis

24 h after the LPS challenge, a total of 36 rats were humanely sacrificed by means of an ethically-administered dose of CO_2_ inhalation. Afterwards, from each group, individual members’ lungs, kidneys, heart, and liver were removed and were subsequently fixed using 10% neutral buffered formalin. 4 μm sections of fixed embedded tissues were cut, mounted on glass slides, deparaffinized and stained with hematoxylin and eosin. Examinations of the aforementioned sections were then carried out by submitting them to empirical observation under a light microscope.

#### Determination of TNF-α and IL-1βby ELISA in rat blood

Blood was withdrawn from the rats from different treatment groups at the fourth and twelfth hours. Then, plasma was separated by centrifugation and added to 96 well plates immobilized with TNF-α or IL-1β antibodies (Abcam, USA). Absorbance values were measured using a microplate reader.

#### Immunohistochemical analysis for TNF-α, COX-2 and iNOS on liver, lungs, heart and kidneys

Immunohistochemical analysis for COX-2, iNOS and TNF-α were conducted using formalin-fixed, paraffin embedded tissue (liver, lung, heart and kidney) sections (4 μm thick) from each treatment group (after CO_2_ euthanasia), adhered on poly-L-lysine pre-coated microscope glass slides. Tissue sections were deparaffinized with xylene, and hydrated with gradient alcohol series, following which endogenous peroxidase was inactivated by keeping it in 3% hydrogen peroxide for 20 min. After the antigen retrieval step using citrate buffer (pH 6.0) at 100^0^ C, the sections were serially stained with diluted primary antibodies (4^0^ C overnight) and HRP conjugated diluted secondary antibody. The final incubation of sections was performed in a chromogen solution containing 3,3’diaminobenzidine (DAB) reagent. Slides were counter-stained with hematoxylin and observed under a light microscope.

#### LC/MS analysis of CETC

In order to facilitate the identification of the bioactive compounds present, CETC was filtered through a 0.22-μm syringe driven filter (Nylon 66) and initially was subjected to LC-MS analysis so as to obtain the Molecular ion mass using Acquity H-Class (Waters) ultra-performance liquid chromatography with BEH C18 column (50 mm × 2.1 mm × 1.7 μm) and a Xevo G2 (Waters) and Mass spectrometer. The mobile phase used was a mixture of acetonitrile and water (10:90) that was delivered at a flow rate of 0.2 ml/min. The electrospray ionization was used in both positive and negative mode with a scan range from m/z 50 to 2000, and the scan time was 9 min. The source temperature and desolvation temperature were 135 and 350 °C, respectively, with the capillary voltage at 4.50 kV. The molecular ion mass which was identified for possible bioactive compound from the LC-MS analysis was further confirmed by detailed LC-MS/MS based analysis using Acquity H-Class (Waters) ultra-performance liquid chromatography with BEH C18 column (50 mm × 2.1 mm × 1.7 μm) and Xevo G2 (Waters) Quadruple Time of Flight (Q-TOF) Mass spectrometer. The compound was further confirmed by the fragmentation pattern analysis.

### Statistical analysis

All data were presented as mean ± SD of three parallel measurements. The statistical analyses were carried out employing one-way ANOVA followed by Tukey post hoc analysis. Any *p*-values less than or equal to 0.05 were considered to be significant.

## Results

### Effects of CETC on the LPS-induced production of NO and PGE_2_ in macrophages

**Fig. 1:**
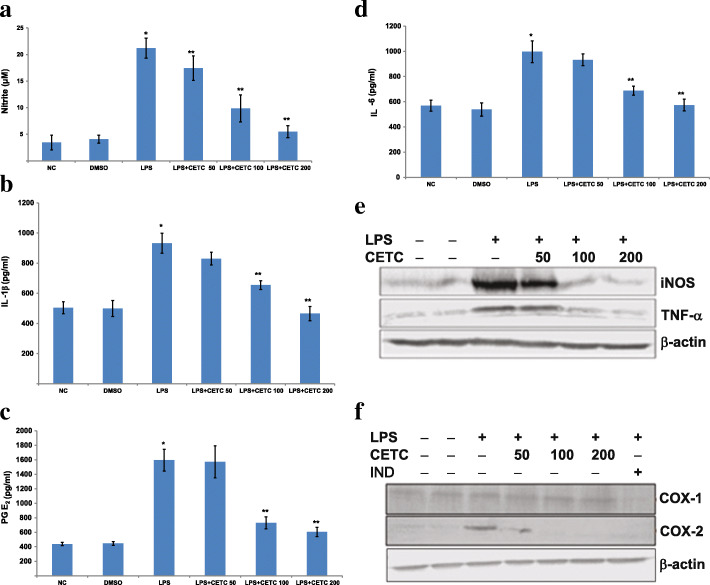
Effects of CETC on the production of pro-inflammatory biomarkers. The treatment of macrophages with CETC resulted in the suppression of (**a**) NO, (**b**) IL-1β and (**c**) PGE_2_ and (**d**) IL-6 synthesis when stimulated with LPS, concentration dependently. Values are mean ± SD, *n* = 3, *p ≤ 0.05 vs. the normal; **p ≤ 0.05 vs. the LPS treated group. **e**-**f** Western blot analyses showed a significant reduction of COX-2, iNOS and TNF-α genes expression when macrophages were pretreated with CETC and stimulated with LPS subsequently. Unlike the indomethacin-treated cells, COX-1 expression of CETC-treated cells remains unaltered. β-actin expression was kept as the internal control. NC-Normal control; D-DMSO treated; IND- cells pre-incubated with indomethacin and simulated with LPS

The levels of nitrite in the macrophage culture supernatant were evaluated as an index of cellular NO production using Griess reagent. The NO secretion was enhanced profoundly by cells treated with LPS alone, as compared to figures exhibited by the unstimulated cells. In contrast, a concentration-dependent reduction in the production of NO was obtained in the case of CETC pre-incubated cells (Fig. 1a). PGE_2_, another potent pro-inflammatory lipid derivative, was also analysed for CETC’s effect on its synthesis in LPS-primed THP-1 cells. As depicted in Fig. 1c, stimulation of macrophages with LPS for 20 h resulted in a marked increase of PGE_2_ secretion as compared to that of the untreated control group of cells. However, CETC inhibited the LPS-induced PGE_2_ production in a concentration-dependent way, suggesting its potential usefulness as an anti-inflammatory agent. With a view to rule out the possibility of the CETC cytotoxic effect, the viability of THP-1 cells cultured with or without LPS (1 μg/ml) in the absence or presence of CETC (25-300 μg/ml) by MTT assay was examined. As shown in the figure, CETC amounts up to 300 μg/ml did not alter cellular viability, a finding which seems to indicate that the reduction of LPS- mediated cytokine secretion in the presence of CETC was not due to cell death (Supplemental Fig. 1).

### Quantification of IL-1β and IL-6 by ELISA in cell culture supernatant

THP-1 cells responded to LPS provocation through a profound increase in the secretion of IL-1β and IL-6, as compared with their basal levels found in the normal control cells. However, the upregulated levels of these interleukins after LPS exposure dropped significantly when cells were pre-treated with CETC in a concentration-dependent manner (Fig. 1b and d).

### Effects of CETC on the expression of TNF-α, iNOS, COX-2 and COX-1 genes in LPS-stimulated THP-1 macrophages

Inflammatory response underlies complex molecular events, one of the most important components of which includes the upregulation of the primary inflammatory genes such as TNF-α, iNOS, and COX-2 in response to bacterial endotoxins and various cytokines [12]. We investigated CETC’s effects on LPS-induced iNOS and COX-2 upregulation in THP-1 macrophages in order to determine whether the reduction of NO and PGE_2_ production was due to a reduced expression of their synthesizing enzymes. It was found that iNOS and COX-2 expressions were upregulated significantly in cells primed with LPS as compared to their expressions observed in unstimulated cells. Interestingly, CETC treatment resulted in a remarkable decrease of the expression of both enzymes under LPS provocation. iNOS and COX-2 proteins showed a significant reduction in the presence of 100 μg/ml CETC. Furthermore, we performed a comparative analysis of the expression of TNF-α in cells pre-incubated with CETC and primed with LPS with rates exhibited by solely-LPS incubated cells. TNF-α concentration was quite high in the lane loaded with cell lysate of LPS-elicited cells as compared to that of the unstimulated cell lysate prepared under the same conditions. This LPS-primed TNF-α induction was shown to be abolished significantly upon CETC pre-incubation (Fig. 1e and f). Since COX-1 gene products are required for the maintenance of homeostasis, anti-inflammatory agents that selectively inhibit the COX- 2 isoform are preferentially valuable. As shown in Fig. 1f, CETC nullified the inductive effect of LPS on COX-2 synthesis sparing the COX-1 production.

**Fig. 2:**
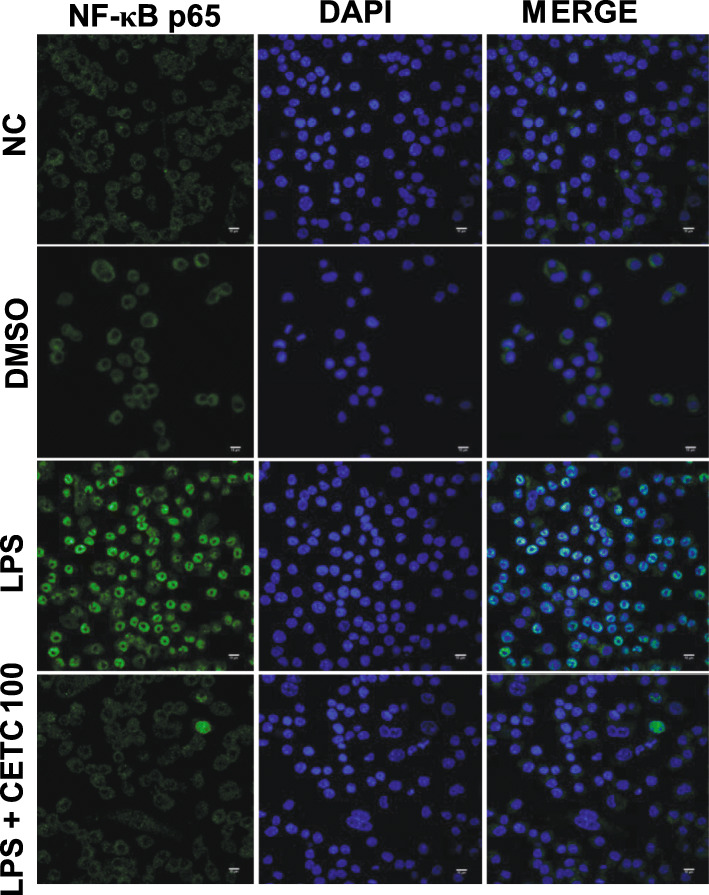
Effect of CETC on LPS induced nuclear translocation of NF-κB in THP-1 macrophages. NF-κB that becomes typically sequestered in the cytoplasm (normal and DMSO control panel) gets concentrated to the nucleus following LPS (1 μg/ml) stimulation (LPS panel), a hallmark of the molecular inflammatory process. But 100 μg/ml CETC pre-treatment actively prevented the nuclear shift of NF-κB which was then found to be distributed all over the cytoplasm (100 μg/ml treated panel) following LPS (1 μg/ml) provocation

### Inhibition of NF-κB activation by CETC in LPS-triggered THP-1 macrophages

Since NF-κB-mediated signalling is crucial for the induction of pro-inflammatory genes, the question regarding the possibility of whether CETC could regulate the translocation of NF-κB from the cytoplasm to the nucleus following LPS stimuli was addressed in the course of research. As a result of our investigations, it was concluded that we had indeed empirically observed a sharp increase in NF-κB levels in the nuclei of LPS alone treated cells in comparison to that in the nuclei of untreated control cells. However, the nuclear accumulation of NF-κB following LPS provocation was inhibited markedly when cells were pre-incubated with CETC 100 μg/ml (Fig. 2).

### Protective effect of CETC on LPS-induced Sepsis in Wistar rats

**Fig. 3:**
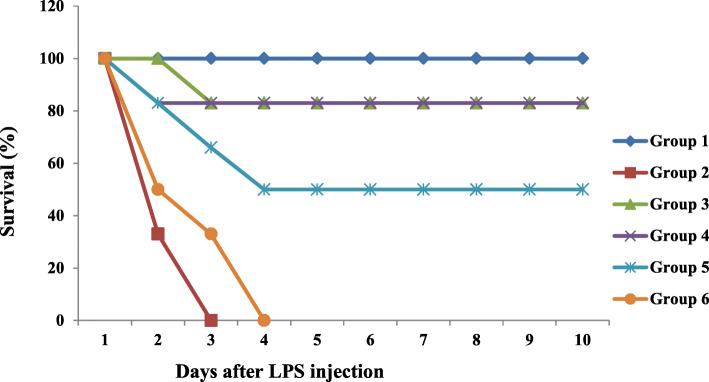
The effect of CETC treatment on the survival rate of rats in a model of LPS-induced septic shock. CETC pre-administration had improved the survival of rats significantly. Survival was monitored for 10 days after LPS (i.p) injection. Group 1- Received saline 10 ml/Kg b.w. orally (Negative control); Group 2- Received saline 10 ml/Kg b.w. orally (Untreated control); Group 3-Indomethacin (10 mg/Kg b.w.) received orally; Groups 4, 5 and 6- Received oral administrations of CETC 500, 250 and 125 mg/Kg b.w. respectively. All rats except Group 1were injected intra-peritoneally with LPS (15 mg/Kgb.w.)

Because CETC was proven to have anti-inflammatory activity by the in vitro study, the effect of CETC administration on a rat model of LPS-induced septic shock was tested. The intra-peritoneal administration of a high dose of LPS (15 mg/kg) resulted in the deaths of all rats from Group 2 within just 2 days. All rats administered with CETC 125 mg/Kg b. w (Group 6) were dead by the fourth day. Survival rates of 50 and 83% were observed with CETC 250 (Group 5) and 500 mg/Kg b.w (Group 4) administered groups of rats respectively. A survival rate of 83% was found in the group pre-administered with indomethacin and primed with LPS (Fig. 3).

### Effect of CETC treatment on plasma IL-1β and TNF-α levels

**Fig. 4:**
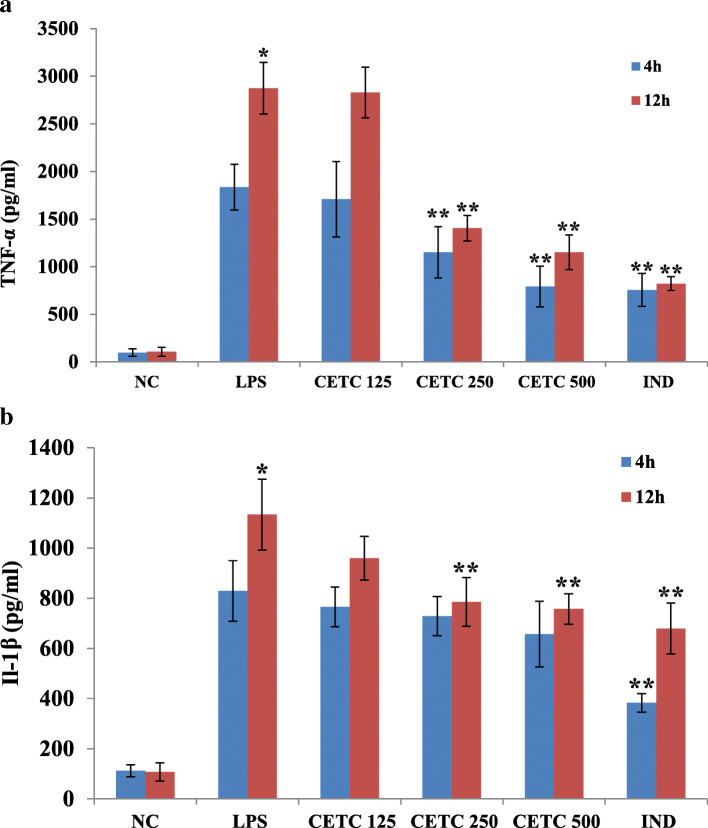
Effects of CETC on the blood levels of (**a**) TNF-α and (**b**) IL-1β.CETC (250 and 500 mg/Kg b.w.) received rats showed a significant reduction in the levels of cytokines as compared to the untreated LPS injected group of rats. Values are mean ± SD, *n* = 6, *p ≤ 0.05 vs. the normal; **p ≤ 0.05 vs. the LPS alone injected group

In the present study, the plasma TNF-α level of LPS injected rats was increased significantly to 1836.34 ± 240.22 pg/ml as compared to the basal TNF level found in vehicle-treated control rats (98.57 ± 39.32 pg/ml). After 12 h of LPS injection, the plasma TNF levels became 2875.24 ± 270.85 pg/ml in untreated LPS-primed rats. Meanwhile, rats pre-treated with CETC 250 μg/ml, the plasma TNF levels were 1150.10 ± 269.78 pg/ml and 1404.33 ± 134.24 pg/ml at 4^th^ and 12^th^h respectively whereas the levels were 791.25 ± 212.89 pg/ml and 1151.38 ± 182.50 pg/ml respectively at 4^th^ and 12^th^ h in CETC 500 μg/ml received group. In addition, the degree of reduction of TNF-α observed in CETC received rats was comparable to that observed in the indomethacin received group – i.e., 756.38 ± 172.06 pg/ml in the 4th h and 822.56 ± 73.20 pg/ml in the 12th h. i.e., pre-treatment with CETC dose dependently attenuated the LPS aided promotion of TNF-α level (Fig. 4a).

Correspondingly, we compared the blood levels of IL-1β in LPS injured, untreated, and CETC pre-administered rats to the vehicle-treated naive rats. LPS-primed, untreated rats showed a sharp rise in IL-1β levels after 4 h as compared with the unstimulated control rats. Strikingly, the plasma levels of this cytokine were observed to be significantly reduced in CETC received rats when challenged with LPS, in a dose-dependent fashion (Fig. 4b).

### Macroscopic evaluation of major organs

**Fig. 5:**
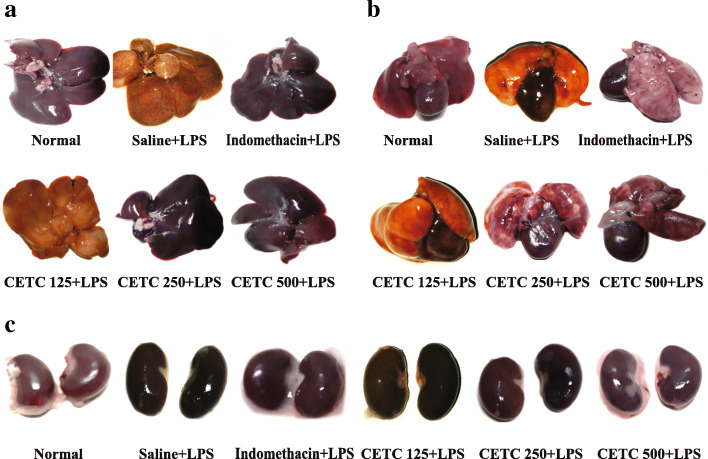
Morphological evaluation of (**a**) Liver (**b**) Heart and Lungs and (**c**) Kidneys of rats in the lethal endotoxemia model

Gross examination of major organs revealed significant lesions after LPS injection as compared to the normal ones. Nonetheless, an improvement was observed upon the treatment with CETC (in a concentration-dependent way) or indomethacin (Fig. 5).

### Histopathological evaluation of major organs

**Fig. 6:**
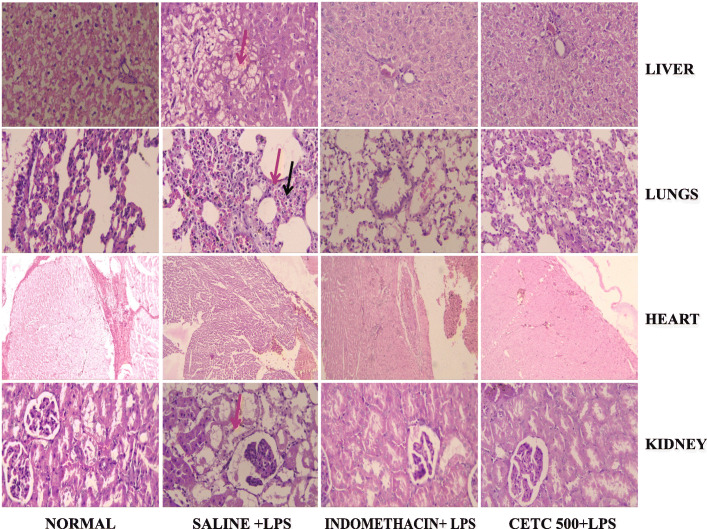
Histopathological evaluation of major organs. The histological lesions were improved remarkably when rats were administered with CETC 500 mg/Kg b.w. before LPS injection. The liver tissue from LPS alone injected rats demonstrated fibrin accumulation and focal necrosis (arrow head). In the case of lung tissue samples observed from specimens representing the LPS-alone-injected group, there was cellular infiltration (black arrow head) and thickening of alveolar septae (red arrow head). Acute tubular necrosis was observed in the tissue sections of the LPS alone injected kidneys of rats (arrow head). Magnification is 200x

The liver tissue from LPS alone injected rats demonstrated ischemic shrinkage, fibrin accumulation and focal necrosis. Additionally, hepatocytes around the portal vein demonstrated vacuolar degeneration and fluid accumulation. In the case of lung tissue samples observed from specimens representing the LPS-alone-injected group, changes observed include congestion, interstitial and alveolar edema, interstitial lymphocytic infiltrate, thickening and fibrosis of alveolar septae, and platelet thrombi in the pulmonary microvasculature. The heart tissue section in this group showed opaque transverse contraction bands in the myocytes near the intercalated disc. Acute tubular necrosis and intra-vascular hemolysis were observed in the tissue sections of the LPS alone injected kidneys of rats. Nevertheless, the severities of these lesions were markedly improved in the CETC received group of rats (Fig. 6).

### Effects of CETC on LPS-induced expression of TNF-α, COX-2 and iNOS in liver, lungs, heart, and kidneys

**Fig. 7:**
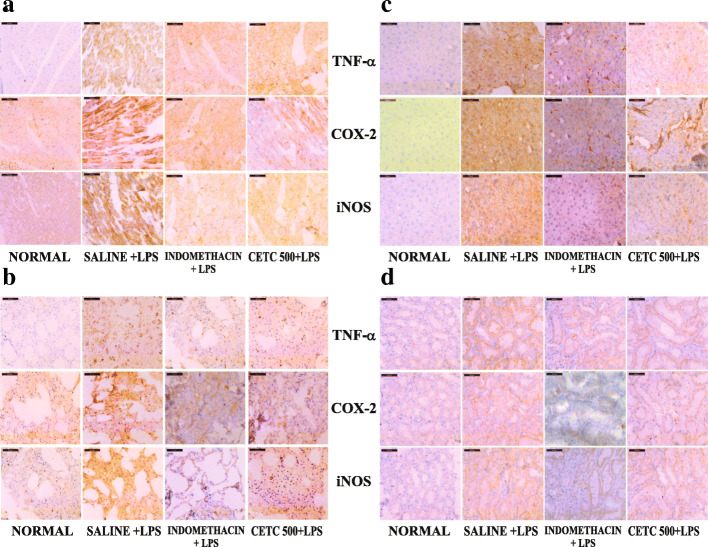
Immuno-histochemical analysis of the expression of pro-inflammatory biomarkers on the tissues of major organs. The expression TNF-α, COX-2, and iNOS were decreased significantly in the tissues **a** Heart, **b** Lungs, **c** Liver and **d** Kidneys of rats pre-administered with CETC 48, 24, 12 and 1 h before LPS injection (i.p. 15 mg/Kg). Scale bar is 100 μm

The objective was to show the reduction of inflammatory changes in the major organs of rats pre-administered with CETC. The tests carried out with the tissues from the LPS group demonstrated a significant immuno-reactivity of TNF-α, COX-2 and iNOS as compared to that of the normal control group whereas the tissue expressions of these pro-inflammatory markers were decreased markedly in rats pre-administered with CETC. Moreover, the degree of reduction was comparable to that observed in the tissues of rats which received the reference drug, indomethacin (Fig. 7).

**Fig. 8:**
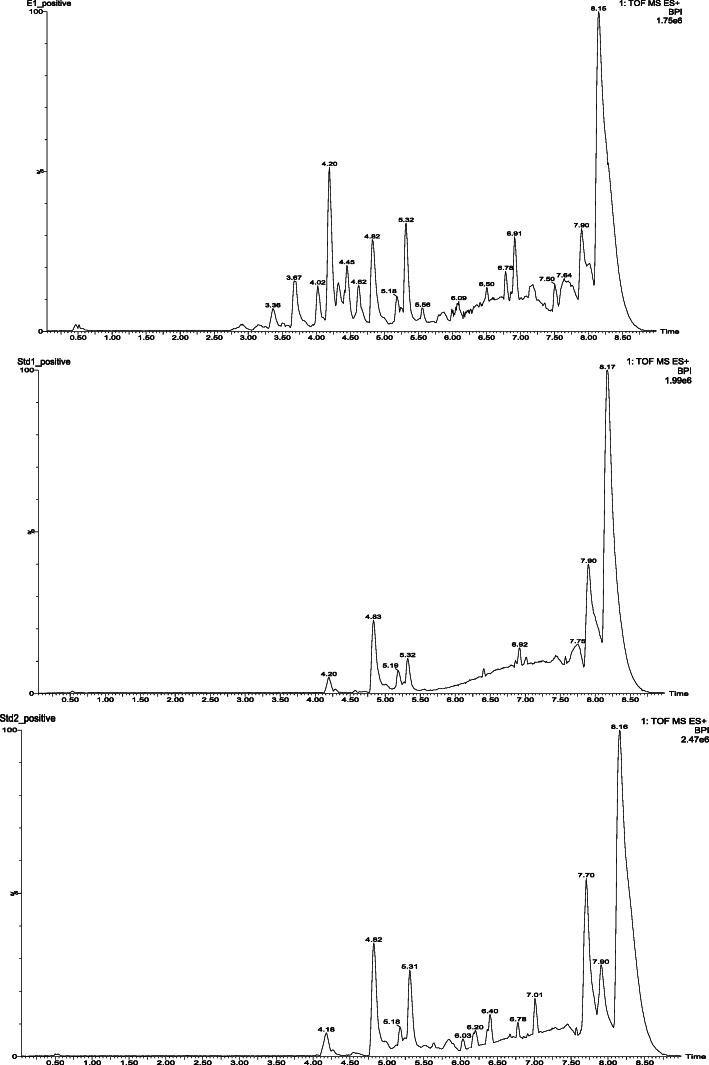
LC/MS analysis of the chloroform extract of *T. cordifolia* (**a**), that of stigmasterol (**b**) and β-sitosterol (**c**)

#### LC/MS analysis of CETC

The phytochemical analysis of CETC was performed using LC/MS analysis and the LC/MS assay detected the presence of stigmasterol and β-sitosterol in CETC (Fig. 8).

## Discussion

In the traditional systems of medicine, polyherbal formulations continue to enjoy a highly-reputed position for the treatment of many disease conditions in which an unregulated inflammatory response amplifies the disease process [13]. However, in order to verify the effectiveness and to elucidate the safety profile of such herbal remedies, traditional knowledge needs to be coupled with scientific research. *T. cordifolia*, one of the most versatile plants with a wide spectrum of pharmacological properties, is a major constituent of Ayurvedic preparations intended for the treatment of rheumatoid arthritis, psoriasis and asthma in which unregulated inflammation aids disease progression. Our previous studies confirmed that CETC contains phytosterols along with other unidentified constituents with the added confirmation of the presence of stigmasterol and β-sitosterol. Many researchers had reported the anti-inflammatory property of phytosterols [14].

In response to an inflammatory stimulus, macrophages secrete effectors such as TNF-α, PGE_2_, leukotrienes, NO, IL-1β and IL-6 [15]. Hence, macrophage cell lines —RAW264.7 and THP-1- stimulated with LPS are commonly used as in vitro inflammation models to study the molecular events of inflammation and for the screening of anti-inflammatory compounds [16, 17]. Initially, we conducted a study to examine whether CETC has anti-inflammatory effects in LPS-stimulated RAW264.7 cells which yielded results with indication that CETC suppressed the iNOS and COX-2 expression and caused a decrease of PGE2, IL-1β and IL-6 secretion with concomitant inhibition of NF-kB activation. Furthermore, CETC produced a significant (*p* ≤ 0.05) suppressive effect on carrageen-induced edema formation. In light of these findings, the present study was undertaken to evaluate CETC’s pharmacological effects on the synthesis of pro-inflammatory biomarkers in LPS triggered by THP-1 macrophages and the effects of CETC in ameliorating or preventing the inflammatory changes in a rat model of endotoxic shock.

COX-2, an isoform induced during inflammatory conditions, catalyses the bisoxygenation and subsequent formation of PGE_2_ from arachidonate. PGE_2_ promotes vasodilation, infiltration of immune cells and edema development at inflammatory sites [18]. NO is a vasodilator and a key regulator of inflammatory response. Various studies have established the crosstalk between PGE_2_ and NO, in which PGE_2_ synthesis is upregulated further in the presence of NO; their combined induction has been directly correlated with the pathogenesis of rheumatoid arthritis, septic shock, Alzheimer’s disease, atherosclerosis and chronic obstructive pulmonary disease [19, 20]. In this regard, the inhibition of COX-2 or iNOS expression offers an excellent strategy to control the severity of chronic inflammation. In the present study, we observed a significant decrease in the production of NO and PGE_2_ which is attributed to the decreased expression of their synthesizing enzymes in the presence of CETC. Moreover, CETC treatment did not significantly alter the expression of COX-1, which is expressed constitutively in many tissues, particularly in the stomach, kidneys and platelets. This is suggestive of its peculiar isozyme-specific inhibition potential.

Pro-inflammatory cytokines such as TNF-α, IL-1β and IL-6 have been empirically confirmed to play a considerable role in regulating the degree of inflammation. TNF-α transmits the inflammatory stimuli to various cell types, potentiating the secretion or expression of NO, PGE_2_, IL-1β, IL-6, collagenases, proteases and cell adhesion molecules. NO and TNF-α act as potent mediators of cytotoxicity. IL-6 regulates the switch between acute and chronic phases of inflammatory response. Previous studies have documented the role of IL-6 and IL-1β deregulation in the pathogenesis of systemic-onset juvenile chronic arthritis, rheumatoid arthritis and psoriasis [21]. In the present study, CETC pre-incubated macrophages showed significantly lower levels of TNF-α, IL-1β and IL-6 upon LPS stimulation as compared to those observed with LPS alone exposed cells, indicating the anti-inflammatory property of CETC more conclusively.

To gain better insight into the molecular basis of CETC-aided cytokine reduction, the degree of NF-κB activation associated with LPS exposure was analysed in macrophages pre-exposed to CETC. NF-κB is a primary regulator of inflammatory response genes and modulates the inducible expression of pro-inflammatory cytokines. This makes NF-κB a suitable target in the effort to find new anti-inflammatory therapeutics [22, 23]. Interestingly, the data obtained in the present study were able to link the reduction of cytokine secretion in the presence of CETC to the reduced levels of nuclear NF-κB for gene activation.

A plethora of data suggested the multifunctional roles played by cytokines which indicates that they are essential to maintain immunological homeostasis and are well implicated in the pathogenesis of different disease states [24]. In large amounts, the release of LPS into the blood stream causes the systemic generation of a potentially-lethal set of pro-inflammatory cytokines and pro-coagulant proteins, leading ultimately to the destruction of the endothelial network, as well as the hypoperfusion of organs, disseminated intravascular coagulation and refractory shock. Thus, suppressing the production of these pro-inflammatory cytokines might be legitimately regarded as a promising therapeutic modality to prevent the development of systemic inflammatory reactions and resulting diseases [25]. Considering the aforementioned finding, the next step was an examination of the effect of CETC pre-treatment on the synthesis of pro-inflammatory cytokines in LPS-primed rats.

The pleiotropic cytokine TNF-α is the most critical mediator of LPS-induced septic reactions. Multiple studies substantiated claims regarding TNF-α’s role in endotoxemic shock and, therefore, the therapeutic target potential in alleviating the associated tissue destruction. Specifically, it was shown that the intravenous injection of TNF-α induced a shock quite similar to septic shock in mice. In addition, TNF-α gene knockouts are proved to be free from severe LPS/D-Galactosamine-induced septic reactions [26]. TNF-α induces systemic activation of complements and procoagulant proteins, mediating the initiation and progression of multiple organ failure. TNF-α and NO are extremely cytotoxic and their enhanced secretion alters organ and cellular physiology at all levels [27, 28]. In the present study, we demonstrated that CETC treatment significantly attenuated the LPS-induced immuno-reactivities of COX-2, iNOS and TNF-α in these organs’ tissues. Most strikingly, cytokines’ plasma levels were also found to be significantly reduced in CETC-received rats when challenged with LPS. It has been validated that the injection of IL-1β alone is enough to reproduce sepsis’s pathological effects in experimental animals, not to mention that it was determined that the pre-treatment of IL-1β receptor antagonists had imparted a significant reduction to the mortality rate of septic patients [29]. Considering these facts, the increased survival rate shown by CETC-received rats can also be attributed to the reduced production of pro-inflammatory cytokines in response to LPS encounter.

Furthermore, the LC/MS analysis confirmed the presence of stigmasterol and β-sitosterol in CETC. Since phytosterol exhibited potent anti-inflammatory activity in various experimental models, the presence of stigmasterol and β-sitosterol might have contributed very significantly to the anti-inflammatory potential of CETC. This result substantiates our previously published results of HPLC analysis further, which also corroborated hypotheses regarding the presence of these phytosterols in CETC [17].

## Conclusion

Interference of inflammatory signalling is now widely recognised as an efficient strategy to prevent or to control pro-inflammatory cytokines’ overzealous action, acknowledged to be the major culprit behind the pathogenesis of chronic inflammatory proliferative disorders. Moreover, compounds or formulations with a peculiarity to selectively downregulate the isoenzymes such as COX-2, if induced only under inflammatory conditions, are more or less free from severe side effects. CETC was found to be very effective in suppressing the production of major LPS inducible cytokines in THP-1 macrophages, without altering the COX-1 synthesis. Furthermore, the inhibitions of TNF-α, iNOS, COX-2, IL-1β, and IL-6 by CETC in endotoxic shock, which are reflected through the increased survival rate of CETC pre-treated rats, are reported here for the first time. Thus, our in vitro findings are in good agreement with the in vivo outcome. In these aspects, it could be expected that CETC might be useful for developing anti-inflammatory drugs with more effectiveness and less toxicity.

## Supplementary Information

**Additional file 1: Supplementary figure 1.** Effect of CETC treatment on the viability of THP-1 cells. THP-1 macrophages were treated with the indicated concentrations of CETC for 24 h. MTT assay was then carried out to determine the percentage cell viability. Results represented here are mean ± SD, *n* = 3. *p ≤ 0.05 denotes significant difference from the normal control group.

## Data Availability

The datasets supporting the conclusions of this article are included in the manuscript. The raw data and materials of the current study are available from the corresponding author on reasonable request.

## Abbreviations

CETC: Chloroform extract of *Tinospora cordifolia*
RPMI: Roswell Park Memorial Institute Medium
SDS: Sodium dodecyl sulphate
DMSO: Dimethyl sulfoxide
PBS: Phosphate buffered saline
PG: Prostaglandin
IL: Interleukin
NO: Nitric Oxide
TNF-α: Tumor necrosis factor-α
COX: Cyclooxygenase
NOS: Nitric oxide synthase
LPS: lipopolysaccharide
ELISA: Enzyme-linked immunosorbent assay
HRP: Horseradish Peroxidase
i.p.: Intra-peritoneal
