# Supplementary figures and images for: Tinospora cordifolia chloroform extract inhibits LPS-induced inflammation via NF-κB inactivation in THP-1cells and improves survival in sepsis
Source: BMC Complement Med Ther. 2021 Mar 20;21:97. doi: 10.1186/s12906-021-03244-y (PMC7980548; doi:10.1186/s12906-021-03244-y)

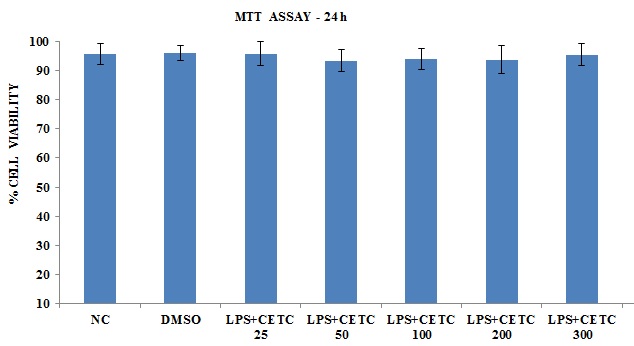


**Supplemental Figure 1**

Supplement: Supplementary file 1 — Additional file 1: Supplementary figure 1. Effect of CETC treatment on the viability of THP-1 cells. THP-1 macrophages were treated with the indicated concentrations of CETC for 24 h. MTT assay was then carried out to determine the percentage cell viability. Results represented here are mean ± SD, n = 3. *p ≤ 0.05 denotes significant difference from the normal control group. [file 12906_2021_3244_MOESM1_ESM.docx]
